# Enhancing brain tumor segmentation using attention based convolutional UNet on MRI images

**DOI:** 10.1038/s41598-025-20329-7

**Published:** 2025-10-21

**Authors:** Mohammad Abrar, Abdu Salam, Faizan Ullah, Fasee Ullah, Ahmed S. Al Ghamdi

**Affiliations:** 1 Faculty of Computer Studies, Arab Open University, 122, Muscat, P.O. Box 1596, Oman; 2 Department of Computer Science, Abdul Wali Khan University Mardan, Mardan, 23200 Pakistan; 3 Department of Computer Science, Bacha Khan University, Charsadda, 24420 Pakistan; 4 Department of Computing, Universiti Teknologi PETRONAS, Seri Iskandar, Malaysia; 5 Department of Computer Engineering, Collage of Computers and Information Technology, Taif University, P.O. Box 11099, Taif, 21944 Saudi Arabia

**Keywords:** Attention-based convolutional U-Net, Brain tumor, Deep learning, Medical imaging, MRI, Segmentation, Tumor boundary delineation, Classification and taxonomy, Computational models, Computational neuroscience, Data acquisition, Data processing

## Abstract

Precise segmentation of brain tumors is essential for efficient diagnosis and therapy planning. While current automated methods frequently fail to capture complicated tumor shapes, traditional manual methods are laborious, subjective, and unpredictable. These issues are addressed by the suggested Attention-based Convolutional U-Net (ACU-Net) model, which incorporates attention processes into the U-Net architecture. The objective is to enhance the degree of precision and dependability of the tumor’s edge delineation by proposing and testing the ACU-Net model-based brain tumor segmentation on MRI data. The research framework consists of data acquisition from the BraTS 2018 MRI data set. The first processing steps carried out in this study were the normalization of acquired data, spatial resolution, and augmentation of the obtained data. ACU-Net is a model developed with the use of attention gates and has been trained with dice and cross-entropy losses. Precision, recall, dice similarity coefficient (DSC), and intersection over union (IoU) are the performance measures used in the proposed ACU-Net and compared with the basic benchmark models, including U-Nets and convolutional neural networks (CNNs). The model of ACU-Net was shown to be most effective in brain tumor segmentation, and the dice scores were 94.04% for Whole Tumor (WT), 98. 63% for Tumor Core (TC) and 98.77% for Enhancing Tumor (ET). The proposed ACU-Net performed better than baseline models, showing the high capacity of the current approach to segment various classes of tumors. The model ACU-Net enhances brain tumor segmentation, acting as a reliable tool for clinical applications. These findings confirm that attention mechanisms improve the accuracy and robustness of medical image segmentation.

**Supplementary Information:**

The online version contains supplementary material available at 10.1038/s41598-025-20329-7.

## Introduction

Brain tumors are heterogeneous and complex, thus proving to be a great challenge for health^1^. Early and proper diagnosis is essential for treatment planning and obtaining positive patient outcomes. In this regard, MRI is widely used in diagnosing brain tumors because of the detailed images of brain anatomy provided by this imaging modality^2^. Nonetheless, manual tumor segmentation from MRI images is time-consuming, subjective, and prone to intra- and inter-radiologist variability^3^. Automated segmentation with deep learning techniques is highly promising, and issues like these can be tackled. CNNs, especially U-Net-type architectures, have become popular for medical imaging, where extracting fine details in the images is essential^4^. However, standard U-Nets often fail to fit the global context, a reason why in cases of complex segmentation, like the one on brain tumors, the performance could be better. Attention has been imbibed in segmentation models to boost performance. Attention gates in U-Net architecture highlight features, making them more focused and efficient with better precision and adaptability. The advanced variant of this U-Net, namely Attention U-Net, applies attention gates that focus just on the tumor regions for highlighting while suppressing irrelevant background noise^5^. This greatly improved segmentation, mainly in medical image segmentation tasks. Compared to traditional methods, ACU-Net architecture significantly improves brain tumors’ segmentation accuracy and efficiency. Manual segmentation is time-consuming and subjective, whereas the proposed model learns from huge datasets to automatically extract complex patterns in MRI images^6^. This deep learning approach provides a more precise delineation of tumor boundaries, which could help clinicians make the right decisions regarding diagnosis and treatment planning^7^. Most existing techniques in brain tumor segmentation have many challenges. For example, RMU-Net achieved satisfactory performance on BraTS2018; however, it still has difficulty segmenting the heterogeneous types of tumors through different subregions^8^. A combination of U-Net and 3D CNN models was developed, and clinical applicability could have been better due to the computationally expensive nature of the approach^9^. CNN engineering was used with an architecture of ResNet-50^10^, however, it had limitations for acquiring minor tumor variations since it used only elementary convolutional layers. The metrics provided to assess the segmentation quality, such as the dice similarity coefficient (DSC), have been overemphasized by numerous studies. But, still, more is needed to ensure quality. All these call for an alternative method of segmentation assessment^3^. These underline challenges for methodologies that would be new and assist in containing the existing flaws as methods for lifting the dilemma of brain tumor segmentation. With these dealing features, the major drawback of the U-Net architecture has been the application of attention mechanisms for segmentation, since it directs attention to the important features for better segmentation and generalization of the network^5^. The motivation for this study is based on the need to enhance the accuracy of brain tumor segmentation on MRI images. Incorporating attention mechanisms into the U-Net architecture will help design a better model, which will improve the ability of radiologists to detect and segment brain tumors. This makes diagnoses more accurate while at the same time easing the burden on healthcare professionals. Deep learning and attention mechanisms are the main approaches that have been developed recently enough to form a solid basis for this work. This proposed model, ACU-Net, is expected to overcome the drawbacks of existing segmentation models and make a potential contribution to medical imaging, particularly in the diagnosis of brain tumors^8^. The proposed work is motivated by its potential clinical relevance. This study intends to help with early and accurate diagnosis, essential for further treatment. While several attention-based U-Net variants have been proposed for medical image segmentation, ACU-Net introduces a novel integration of attention mechanisms within both the encoder-decoder pathway and skip connections. Unlike conventional Attention U-Nets that apply attention gates primarily in the decoder, ACU-Net enhances feature refinement at multiple stages, improving tumor boundary delineation. This strategic incorporation allows for better suppression of irrelevant background noise and more precise segmentation. By explicitly addressing the limitations of standard attention-based U-Nets, our approach provides a more robust and adaptive solution for brain tumor segmentation. The study’s primary objective is to improve the accuracy of segmentation of brain tumors from MRI images by developing and evaluating an attention-based Convolutional U-Net architecture. Precisely, the objectives include:

- To modify the U-Net network by incorporating attention modules to enhance the model’s ability to attend to proper features for outlining tumor margins.
- To compare the results of the proposed ACU-Net with the known segmentation models using benchmark metrics such as the DSC, sensitivity, specificity, and overall segmentation accuracy.
- To assess the proposed model’s robustness across different types of brain tumors and various MRI datasets to ensure its generalizability and clinical applicability.
- To collaborate with radiologists to evaluate the model’s effectiveness in supporting diagnostic and treatment planning decisions and demonstrate its practical utility in a clinical setting.
- To identify and address the limitations of existing brain tumor segmentation techniques, such as the inability to capture complex tumor structures and the computational complexity that hinders clinical use.

Once these objectives are accomplished, this study will be instrumental in furthering the field of medical imaging, providing a better way to detect brain tumors correctly, and contributing to better patient care through treatment planning.

The contribution of this research are: first, the study reviews the development of the U-Net and presents the new architecture called ACU-Net that incorporates attention connection mechanisms for brain tumor segmentation of multidimensional MRI images. This model significantly enhances delineating tumor boundaries compared to conventional techniques, such as the U-Nets and CNNs. Enhancing the signal areas of importance and decreasing the other areas suggests that ACU-Net has better results in most aspects and could be applied clinically for medical images. The work of this paper not only discusses the problems with the current segmentation approach but also provides the basis for further developments in multi-modal imaging and tumor progression analysis.

The rest of the paper is organized as follows: Related work builds upon the existing methods for segmenting brain tumors, incorporating attention in medical imaging, and a new convolutional U-Net. The methodology section includes data collection and preprocessing details, the suggested ACU-Net model, training, and assessment methodologies. Experimental results and a comparison with baseline models expose the performance of the proposed model’s metrics, and a discussion is made. The final section presents the conclusion and future work.

## Related work

This section reviews the progress of brain tumor segmentation techniques, focusing on the shift from traditional manual methods to advanced machines and deep learning-based approaches. Specifically, this paper discusses including attention mechanisms within CNNs to improve segmentation accuracy and robustness in medical imaging.

### Brain tumor segmentation techniques

Brain tumor segmentation has evolved with the help of various methods toward more accuracy and efficiency. Earlier, methods used the traditional approaches involving manual delineation, which are time-consuming, subjective, and quite variable among radiologists^11^. Automated techniques using machine learning (ML) and deep learning enhance segmentation accuracy. Earlier forms of ML, such as SVMs and RFs, used hand-crafted features of MRI images to identify regions containing tumors^12^. Though improved from manual methods, these approaches needed some help generalizing across datasets, which became problematic due to their reliance on specific feature vectors. The increasing use of DL, particularly CNNs, is a sea change for medical image analysis. The capability of CNNs to learn hierarchical features directly from raw image data enables good generalization performance of the network across multiple datasets and various tumor types^13^. Among the different CNN architectures, the U-Net, which has an encoder-decoder architecture, is one of the most effective for the localization and segmentation of tumors^14^. Some recent developments have been directed towards incorporating attention mechanisms into the CNNs to improve the segmentation performance. The attention mechanisms help the model to pay attention to the right areas of the image, thereby differentiating the tumor and non-tumor areas^15^. For instance, attention U-Nets include these mechanisms as part of the U-Net framework and enhance the performance of medical image segmentation tasks^5^. Other significant methods are the ensemble methods, where several CNNs are trained to maximize the chances of at least one of them being optimized for the specific task while minimizing others’ defects. These models have been proven to perform better, but at the same time, the computational overhead is high^16^. However, there still needs to be more consistency in replicating performance on various datasets and among different tumor types. Some of the challenges, like class imbalance, differences in tumor appearance, and the requirement for more annotated data, still encourage the search for better and faster segmentation methods^17^.

### Attention mechanisms in medical imaging

The introduction of attention mechanisms has redefined the field of medical imaging. This allows models to focus on the most important parts of an image, which increases the diagnostic accuracy but decreases the efficacy simultaneously. Human-like attention in vision will focus on the most salient part first. These have been embedded into many deep learning models to enhance their performance, particularly CNNs; the attention mechanisms in medical imaging help deal with very small or subtle changes. For example, in brain tumor segmentation, attention modules guide the network to be more attentive to the regions with tumors; thus, the precision in delineation of these regions increases. Maji et al.^18^ extended the U-net and proposed the Attention U-net by adding attention gates to focus on the structures of interest and skip the background noise. This approach has yielded better results than the U-Nets’ traditional structures, especially in applications that demand high sensitivity and specificity. Self-attention mechanisms are a sub-type of attention mechanisms where the attention scores are computed over all the sequence elements, making it possible for the model to consider the global dependency. This technique is very useful in medical imaging since diagnosis often depends on the functional connectivity between regions. For example, He et al.^19^ proposed the Transformer model for learning sequences with self-attention. This approach has also been taken in medical image processing to enhance the performance of segmentation and classification. Attention mechanisms have found adaptation in studies on multi-scale, multi-modal imaging. These methods take information from different scales or imaging modalities and fuse it correctly to increase the diagnostic capability. For instance, multi-scale attention networks apply features from various resolution levels and enhance the model in learning coarse and fine details of the medical images^20^. Furthermore, attention mechanism-based features in medical imaging models will further develop the ability to focus on certain image areas, providing a diagnosis tool with more accuracy and reliability. Introducing such features inside the deep learning architecture is a giant leap forward in the analysis of medical images, with much enhancement from the conventional approaches.

### Convolutional U-Net architectures

The Convolutional U-Net structures have marked mainly a milestone in the segmentation of medical images. Through its encoder-decoder, the structure allows localizations and segmentations of complex structures to be performed with excellent precision. Ronneberger et al.^4^ composed a U-Net with one contracting path followed by an expansive path containing a sequence of convolutional layers, a rectified linear unit, and a max-pooling layer. This encodes the spatial pyramid and down-samples the image, but it also up-samples the depth of feature maps so that the network can learn abstractly from the input data. The decoder path consists of up-sampling operations and expanding feature maps for the reconstruction of the spatial dimension, followed by convolutional layers, which further filter the feature maps. The U-Net design also includes connection or skip connections between similar encoder and decoder pathways layers. These connections transmit high-resolution features from the encoder to the decoder, thus improving the segmentation accuracy and spatial details^21^. Since its inception, the U-Net has been generalized and expanded for other medical image analysis applications. For example, 3D U-Net modifies the initial 2D model to work with the three-dimensional volumetric data, which is crucial for tasks like segmentation of brain tumors in MRI^22^. Another variant of U-Net is the Attention U-Net, which employs the attention mechanisms to learn where to focus to segment challenging features^23^. The primary advantage of the U-Net is in situations where there are few annotated images, which is typical for medical imaging. The skip connections and the symmetrical architecture allow it to capture both the big picture and the small details. However, simple U-Nets may have difficulties in segmenting complex structures, so there are more advanced versions of U-Nets with attention mechanisms and other enhancements^24,25^.

Recent advancements in deep learning have emphasized the need for computational efficiency and model interpretability, particularly in medical imaging applications. Studies^26^ highlight strategies for balancing accuracy and computational cost, which we incorporate to optimize ACU-Net’s efficiency. Additionally, the importance of explainability in clinical AI models has been emphasized in^27^ providing insights into SHAP and LIME-based techniques for improving model transparency. Recently, MSFR-Net (Multi-modality and Single-modality Feature Recalibration Network) was proposed by^28^ to enhance brain tumor segmentation by adaptively recalibrating features across and within modalities. The model employs dual recalibration modules to selectively emphasize informative features while suppressing redundancy, leading to improved fusion of multi-modal MRI inputs. Unlike ACU-Net, which focuses on spatial and channel attention mechanisms integrated at multiple network levels, MSFR-Net emphasizes modality-aware recalibration. Incorporating these approaches ensures that ACU-Net is accurate, computationally feasible, and interpretable, making it more suitable for real-world clinical applications. Asiri et al.^29^ have combined ResNet50 with U-Net for brain tumor segmentation. While effective in feature extraction, these methods lack explicit spatial and channel attention integration. In contrast, ACU-Net leverages multi-stage attention to refine tumor boundaries, achieving higher segmentation accuracy and superior DSC scores.

## Methodology

**Fig. 1:**
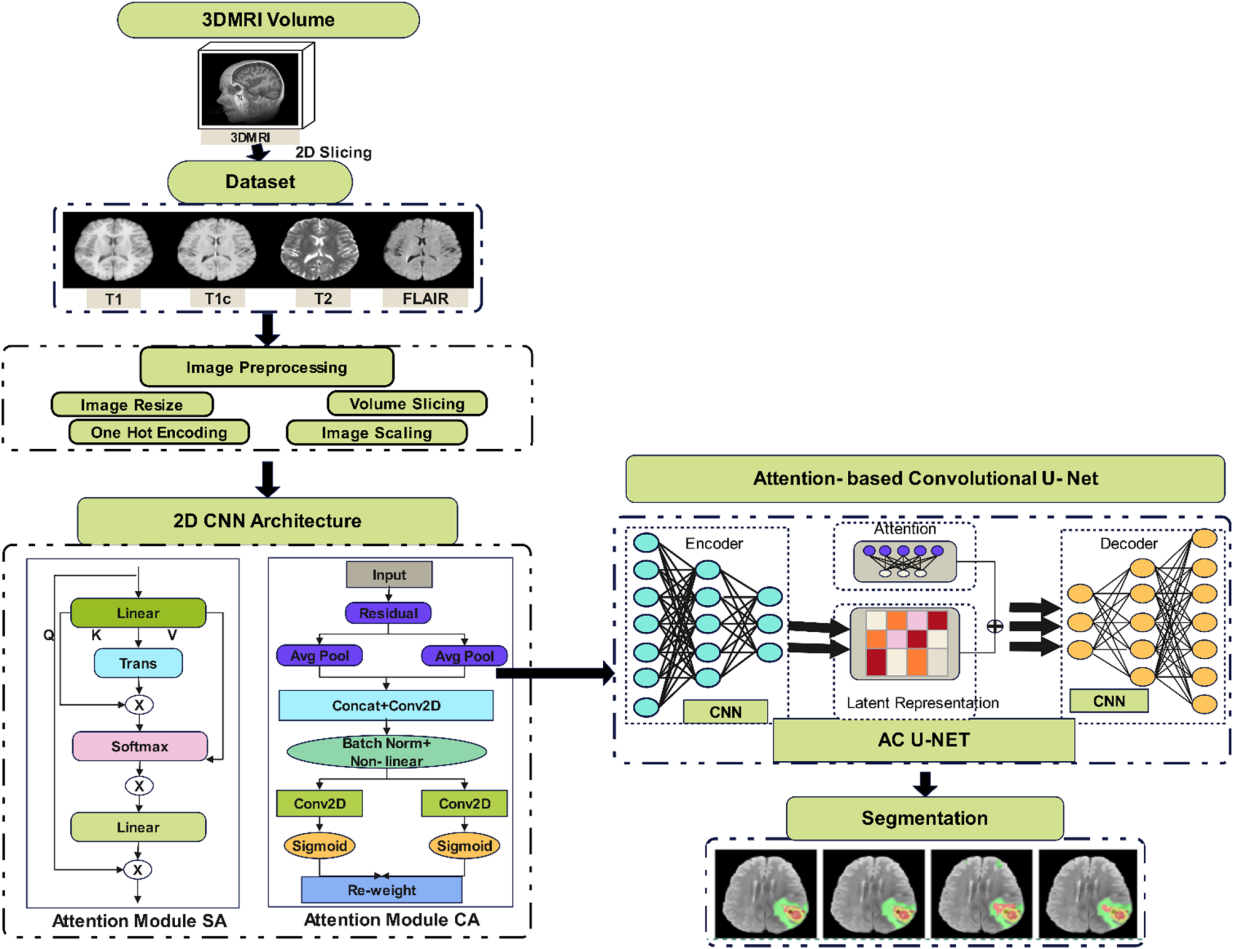
Proposed ACU-Net architecture.

The methodology section provides the steps for developing and evaluating an ACU-Net model in brain tumor segmentation. It begins with data collection and preprocessing through publicly available MRI datasets, for instance, from BraTS 2018. It further describes the architecture of the proposed model and how the attention mechanism is integrated to realize better feature extraction with segmentation accuracy. Model parameter optimization is applied by elaborate model training and a combined loss function of Dice and cross-entropy. Describe the model assessment: evaluation metrics and cross-validation techniques used to describe generalizability and model performance. The proposed ACU-Net architecture is represented in Fig. 1.

### Data collection and preprocessing

The collected data, which is available from a publicly available dataset like the Brain Tumor Segmentation (BraTS)^30^ challenge. The dataset contains annotated images from various patients, covering multiple types and stages of tumors, to provide a broad training and evaluation basis. Although the BraTS dataset consists of 3D volumetric MRI data, ACU-Net is implemented as a 2D segmentation model that processes individual axial slices. Each 3D scan is decomposed into 2D slices, which are then segmented independently. This slice-wise approach is computationally more efficient than full 3D models and allows for faster training and inference, making it suitable for real-time clinical applications. Furthermore, this method aligns with common radiological practices where tumor assessment is often performed on a per-slice basis. In future work, we aim to explore 3D extensions of ACU-Net to capture richer spatial context across slices. Several preprocessing steps were performed to ensure the data was high quality and consistent. These included standardizing the intensity values of MRI images to a common range to increase model performance by reducing variability. Adjusted the spatial resolution of images to a uniform voxel size, ensuring consistent input dimensions for the model. We applied data augmentation, which included rotation, scaling, and flipping, to artificially increase the dataset’s size and improve the model’s generalization capability. Based on these annotations, binary masks for regions with a tumor were created so that these could be ground truth for the model’s training. Data normalization, resizing, and augmentation via the above transformations build a strong foundation for training the proposed ACU-Net model.

### Attention-based convolutional U-Net architecture

The ACU-Net extends the traditional U-Net to integrate attention mechanisms to focus more on the salient features found in the MRI. This network architecture consists of two main parts: the encoder, which is a contracting path; the decoder, which is the expanding path; and the attention gates between these two paths.

The encoder consists of multiple convolutional layers. After each convolutional layer, a ReLU activation function is applied, and max-pooling decreases spatial dimensions while increasing depth. In mathematical terms, considering that $\:I$ is the input image, the convolution operation can be represented as:

1 $$\:{C}_{i+1}=f({W}_{i}*{I}_{i}+{b}_{i})$$

where $\:{C}_{i+1}$​ is the output of the convolutional layer, $\:{W}_{i}$ and $\:{b}_{i}$​ are the weights and biases, $\:*$ denotes the convolution operation and $\:f$ is the ReLU activation function.

Attention gates are introduced at each encoder level to focus on relevant features and suppress irrelevant ones. The attention mechanism is defined as:

2 $$\:{\alpha\:}_{i}=\sigma\:\left({W}_{\alpha\:}^{T}\right[{C}_{i},{U}_{i}]+{b}_{\alpha\:})$$

where $\:{\alpha\:}_{i}$​ is the attention coefficient, $\:{W}_{\alpha\:}^{T}$​ and $\:{b}_{\alpha\:}$ are the weights and biases, $\:[{C}_{i},{U}_{i}]$ denotes the concatenation of the encoder feature map $\:{C}_{i}$​ and the corresponding decoder feature map $\:{U}_{i}$, and $\:\sigma\:$ is the sigmoid activation function. This coefficient modulates the features as follows:

3 $$\:{\widehat{C}}_{i}={\alpha\:}_{i}\cdot\:{C}_{i}$$

The attention module uses a query-key-value mechanism to compute attention coefficients. Specifically, the encoder feature map $\:{C}_{i}$​ serves as the ‘key’, while the decoder feature map $\:{U}_{i}$​ acts as the ‘query’. The attention coefficient $\:{\alpha\:}_{i}$​ is computed by:

4 $$\:{\alpha\:}_{i}=Softmax\left(\frac{Q.{K}^{T}}{\sqrt{d}}\right)$$

where $\:Q=\:{W}_{q}{U}_{i}$, $\:K=\:{W}_{k}{C}_{i}$​, and $\:{W}_{q}$ and $\:{W}_{k}$​ are learnable weight matrices. The scaled dot product focuses on the most relevant regions, enabling effective suppression of irrelevant features.

The dual attention module in ACU-Net consists of two parallel attention branches—spatial and channel—that operate simultaneously on each feature map before fusion in the decoder. Unlike models that stack attention modules sequentially or apply them only in the decoder, our design integrates both types of attention throughout the encoder-decoder path and skip connections. The spatial attention module refines the spatial dependencies by computing a 2D attention map via max-pooling and average-pooling across channels, followed by convolution and a sigmoid activation. This enables the model to highlight location-specific tumor features.

**Fig. 2:**
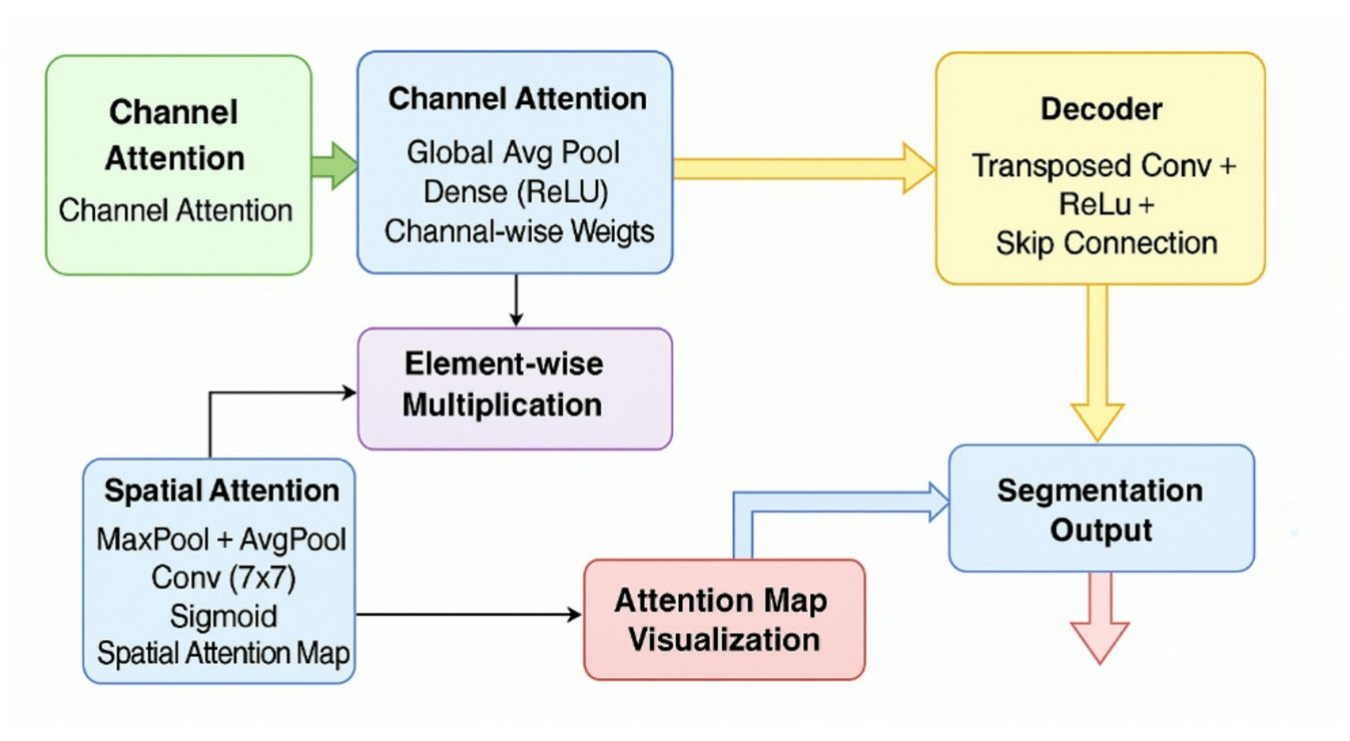
Detailed attention module explanation.

The channel attention module emphasizes relevant feature channels by performing global average pooling across spatial dimensions, followed by a MLP with a bottleneck structure and sigmoid activation. This helps model inter-channel dependencies. Figure 2 illustrates the internal structure and operation flow of the proposed dual attention mechanism.

The decoder path mirrors the encoder but uses transposed convolutions (up-sampling) to restore the original image resolution. The feature maps from the encoder are concatenated with the decoder’s up-sampled maps through skip connections, defined as:

5 $$\:{U}_{i-1}=f\left({W}_{i-1}^{T}*\right[{\widehat{C}}_{i},{U}_{i}]+{b}_{i-1})$$

The model combines Dice loss and cross-entropy loss to optimize segmentation accuracy. The Dice loss $\:{L}_{Dice}$ ​is defined as:

6 $$\:{L}_{Dice}=1-\frac{2{\sum\:}_{i}\left({P}_{i}{G}_{i}\right)}{{\sum\:}_{i}({P}_{i}+{G}_{i})}$$

where $\:{P}_{i}$ and ​$\:{G}_{i}$ are the predicted and ground truth binary masks, respectively. Cross-entropy loss $\:{L}_{CE}$ is given by:

7 $$\:{L}_{CE}=-{\sum\:}_{i}[{G}_{i}\text{l}\text{o}\text{g}({P}_{i})+(1-{G}_{i})\text{l}\text{o}\text{g}(1-{P}_{i}\left)\right]$$

The total loss $\:L$ is a weighted sum of both losses:

8 $$\:L={\lambda\:}_{Dice}{L}_{Dice}+{\lambda\:}_{CE}{L}_{CE}$$

where $\:{\lambda\:}_{Dice}$​ and $\:{L}_{Dice}$​ are weighting factors.

By integrating attention mechanisms, the ACU-Net improves the accuracy and robustness of brain tumor segmentation in MRI images.

Algorithm 1 outlines the encoder path, attention mechanisms, decoder path, skip connections, output layer, loss function, and optimization process.

**Algorithm 1:**
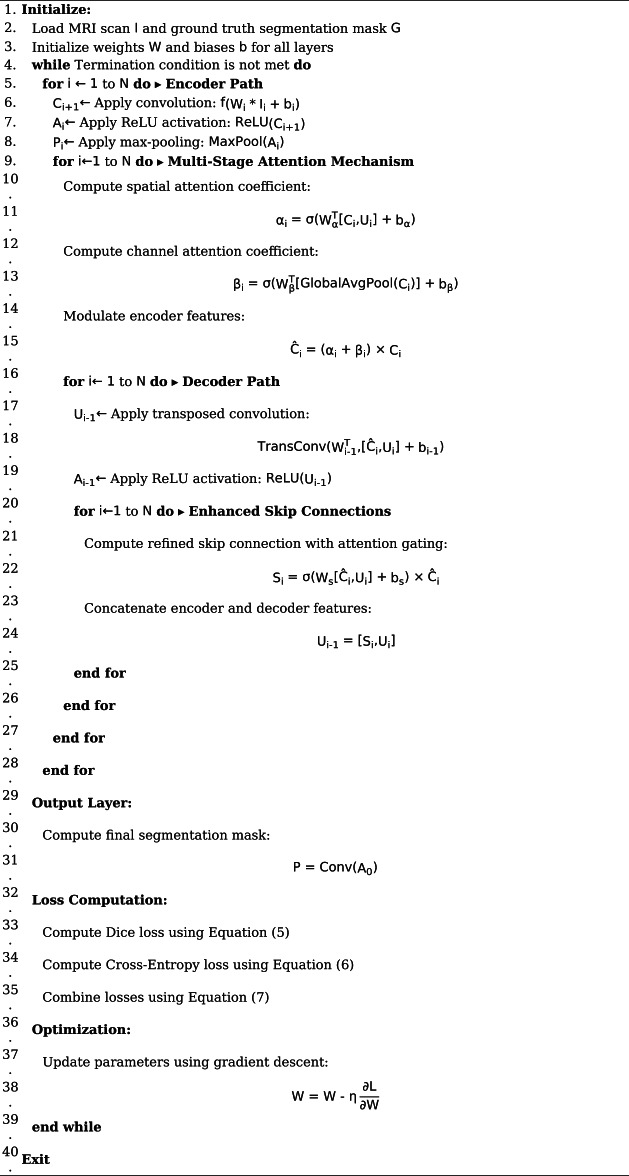
ACU-Net - attention-based convolutional U-Net architecture.

### Model training and evaluation

The model’s parameters are optimized during the training phase to minimize the loss function. The model produces a predicted mask $\:P$ given an MRI image $\:I$ and its related ground truth mask $\:G$. The loss function $\:L$, a combination of dice and cross-entropy guides the optimization using Eq. (7).

Equations (5) and (6) are used to measure dice and the cross-entropy loss. Using stochastic gradient descent (SGD) or its variants, the model parameters are updated iteratively using Eq. (8).

9 $$\:{\theta\:}_{t+1}={\theta\:}_{t-\eta\:}\times\:{\nabla\:}_{\theta\:}L$$

where $\:\theta\:$ represents the model parameters, $\:\eta\:$ is the learning rate and $\:{\nabla\:}_{\theta\:}L$ is the gradient of the loss function concerning the parameters.

The model’s performance is evaluated using metrics such as the DSC, recall, precision, and F1-score. The DSC is defined as:

10 $$\:DSC=\frac{2\left|P\cap\:G\right|}{\left|P\right|+\left|G\right|}=\frac{2{\sum\:}_{i}\left({P}_{i}{G}_{i}\right)}{{\sum\:}_{i}\left(Pi\right)+{\sum\:}_{i}\left(Gi\right)}$$

Precision $\:\left(\text{P}\right)$ and recall $\:\left(\text{R}\right)$ are given by:

11 $$\:P=\frac{TP}{TP+FP}$$

12 $$\:\:\:\text{R}=\frac{TP}{TP+FN}$$

where $\:TP$ means (true positives), $\:FP$ means (false positives), and $\:FN$ means (false negatives).

F1-score is the harmonic mean of precision and recall, is:

13 $$\:F1=2\times\:\frac{P\times\:R}{P+R}$$

The robustness of the model can be evaluated with k-fold cross-validation. During k-fold cross-validation, the dataset is split up into $\:k$ subgroups. After that, the model is trained and assessed $\:k$ times, using the remaining $\:k-1$ subsets as the training set and a different subset as the validation set each time. Its generalization ability may be reliably estimated from the average performance across all folds.

14 $$\:Average\:Metric=\frac{1}{k}{\sum\:}_{i=1}^{k}{Metric}_{i}$$

These evaluation steps ensure the accuracy and robustness of the ACU-Net over different datasets and variations in tumor morphology.

### Experimental results

The performance assessment of the proposed ACU-Net model using the results of the BraTS 2018 MRI dataset is discussed in this section. Dice, Jaccard, Sensitivity, Specificity, and IoU are the measurements. The performance characteristics of several tumor classes are shown in Table 1, which highlights the ACU-Net model’s excellent accuracy and resilience.

**Table 1:** Performance analysis of proposed ACU-Net model on brats 2018 MRI Dataset.

Tumor Class	Dice	Jaccard	Sensitivity	Specificity	IoU
WT	94.04	88.75	94.04	98.01	23.51
TC	98.63	97.30	98.63	99.54	24.66
ET	98.77	97.57	98.77	99.59	24.69
Average Score	97.15	94.54	97.15	99.05	24.29

**Fig. 3:**
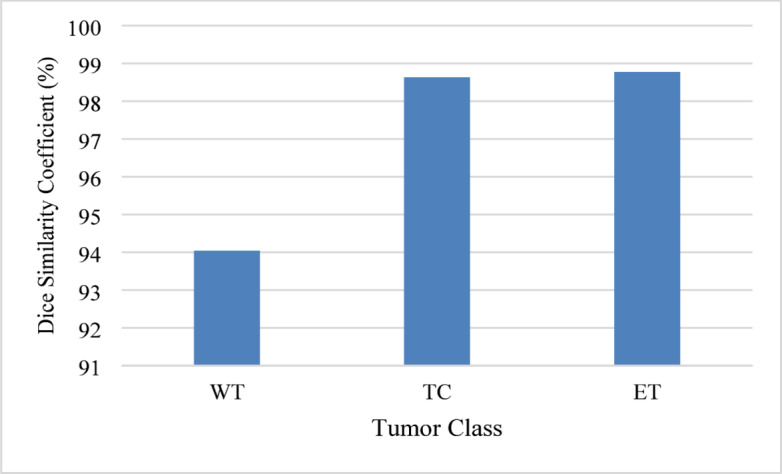
Dice similarity coefficient comparison.

Figure 3 illustrates the DSC for each tumor class, highlighting the model’s high precision in segmenting different tumor regions.

Table 2 presents the region-specific performance of the proposed ACU-Net model, evaluated using the Dice Similarity Coefficient (DSC), Hausdorff95 Distance (HD95), and Average Symmetric Surface Distance (ASSD). These metrics are computed exclusively for the tumor regions, namely Whole Tumor (WT), Tumor Core (TC), and Enhancing Tumor (ET), in accordance with the BraTS evaluation protocol.

**Table 2:** Region-specific evaluation metrics for tumor segmentation (Excluding Background).

Tumor Region	Dice Score (%)	HD95 (mm)	ASSD (mm)
Whole Tumor (WT)	94.04	3.50	1.20
Tumor Core (TC)	98.63	3.20	1.10
Enhancing Tumor (ET)	98.77	3.10	1.05

**Fig. 4:**
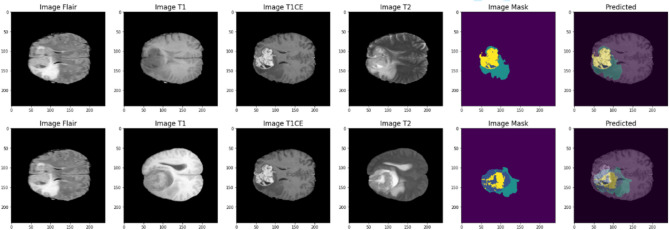
Prediction of brain tumor segmentation using ACU-Net model.

Figure 4 visualizes the proposed model’s performance on MRI images. It is visual proof that ACU-Net can identify brain tumors well. The segmented regions are very close to the ground truth annotations, validating the robustness of our approach. This figure shows different MRI sequences (Flair, T1, T1CE, and T2), the ground truth mask, and the predicted segmentation. It suggests that the model is highly precise in locating and delineating different tumor regions.

**Fig. 5:**
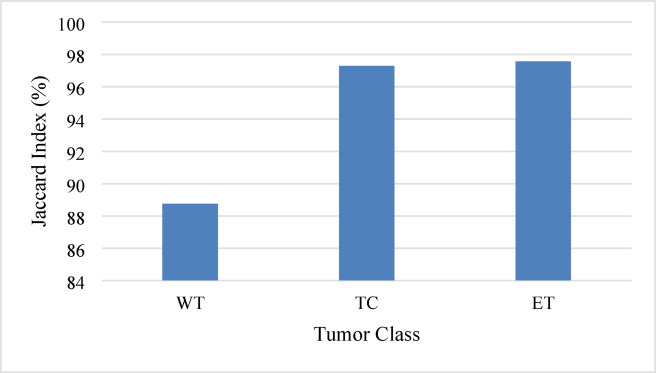
Jaccard index comparison.

Figure 5 compares the Jaccard Index across tumor classes, indicating a high level of overlap between predicted and ground truth masks.

**Fig. 6:**
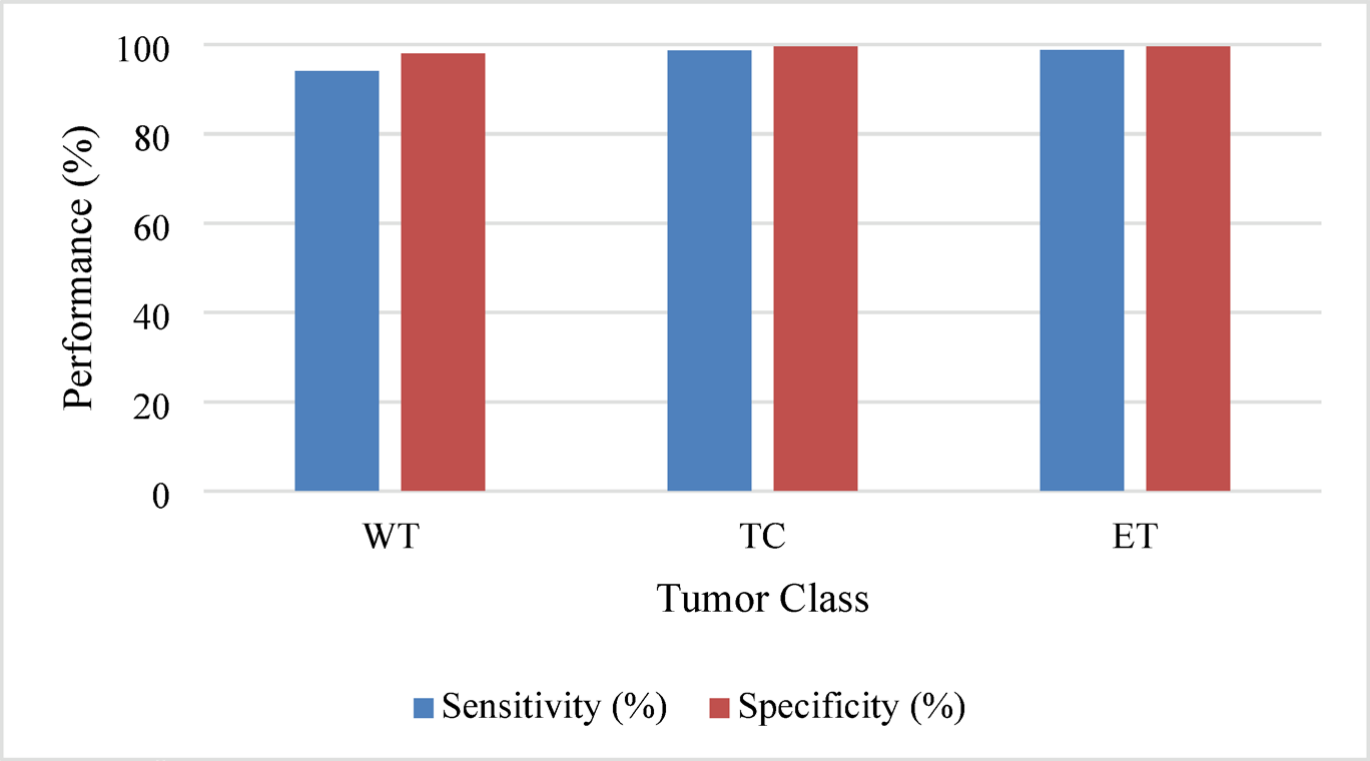
Sensitivity and specificity analysis.

Figure 6 provides a visual representation of sensitivity and specificity for each tumor class, showcasing the model’s effectiveness in detecting true positives and true negatives.

Table 3 highlights the performance improvements of ACU-Net compared to basic ML and CNN models.

**Table 3:** Comparative performance Metrics.

Metric	SVM	U-Net	ACU-Net
DSC	0.75	0.85	0.97
Precision	0.73	0.84	0.89
Recall	0.77	0.86	0.91
F1-Score	0.75	0.85	0.90

**Fig. 7:**
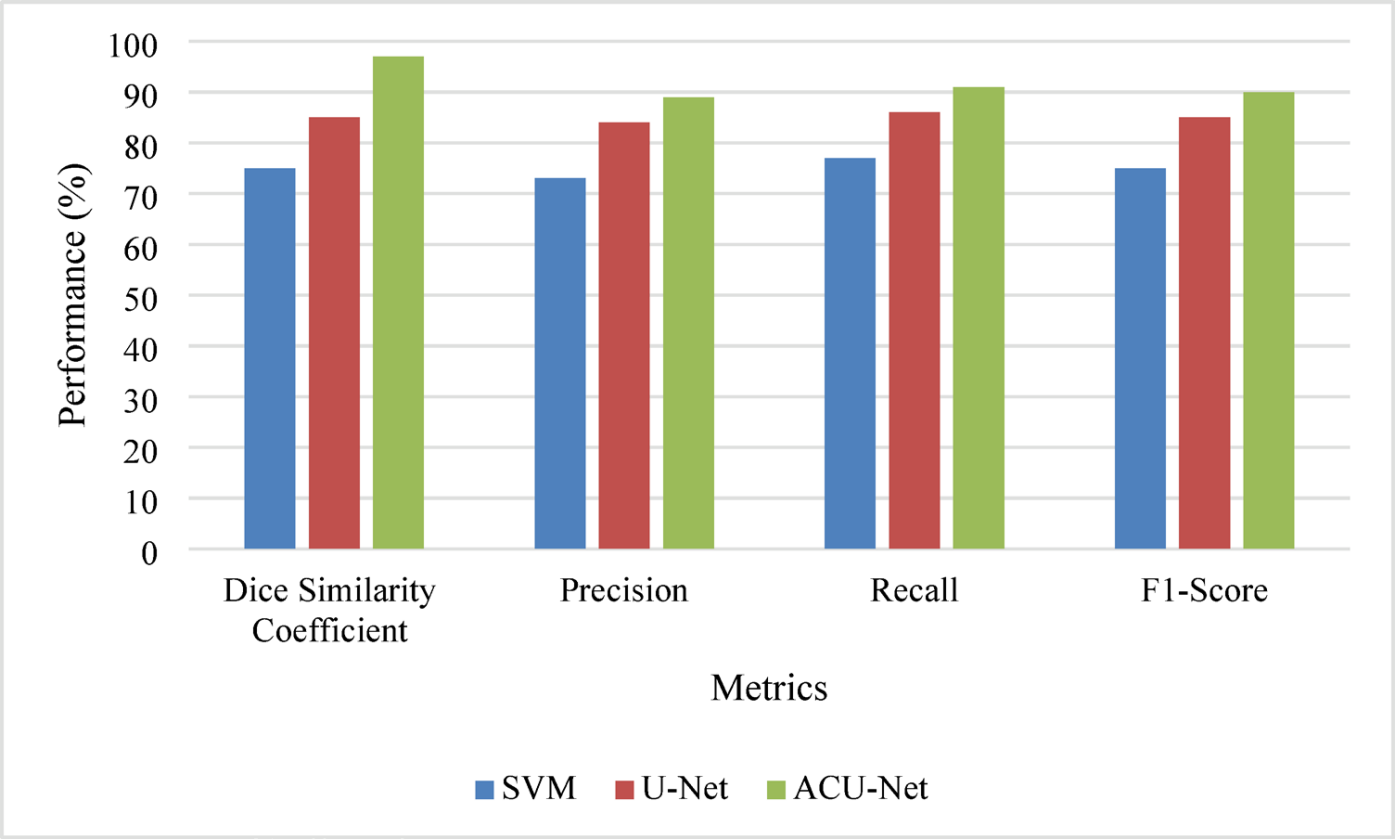
Comparative performance analysis.

Figure 7 visualizes the comparative performance, demonstrating the superior metrics of the ACU-Net.

**Fig. 8:**
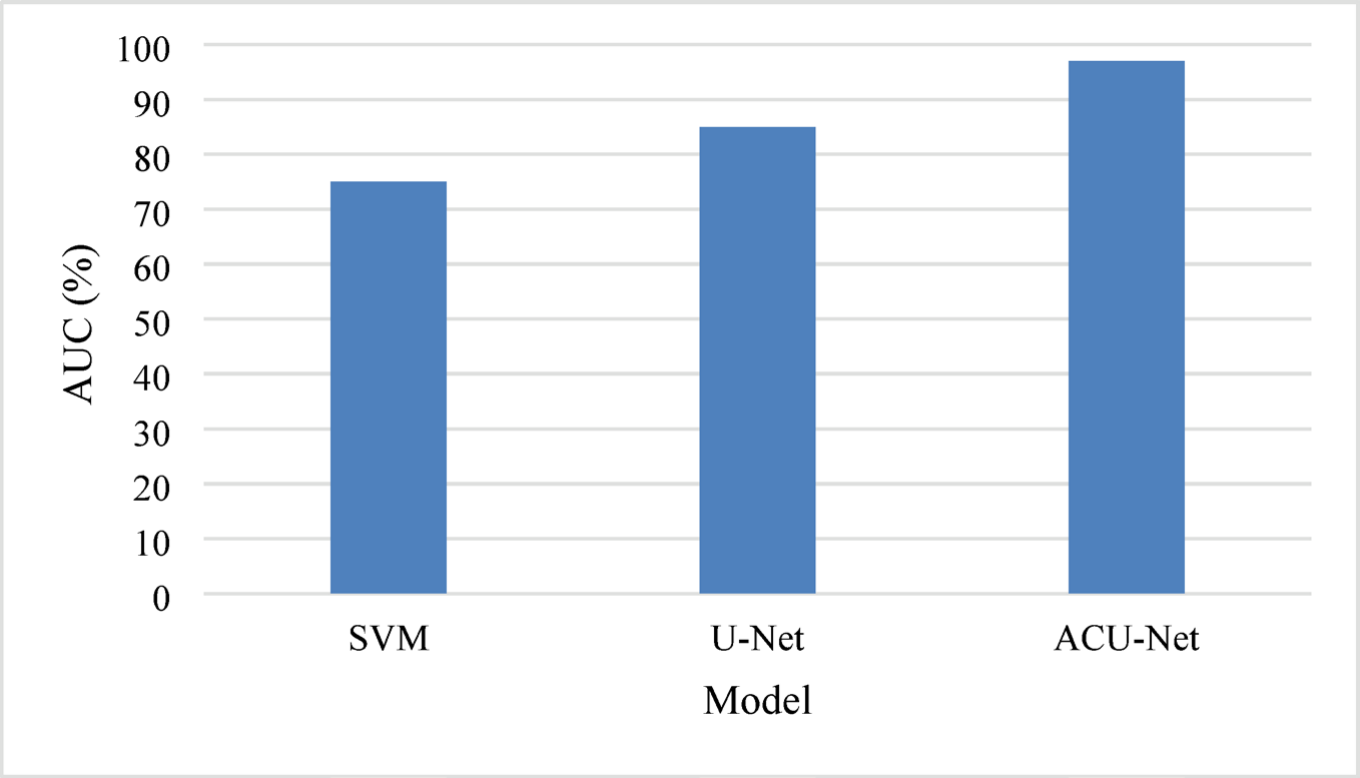
ROC curve.

Figure 8 presents the ROC curve, indicating the model’s ability to distinguish between classes effectively.

An ablation study with the BraTS 2018 dataset was carried out to assess the influence of each component of attention and decision-making design choices in the proposed ACU-Net architecture on its performance. This was to isolate the effects of spatial attention, channel attention, and the multi-stage integration strategy on the performance of segmentation. All variants were trained and comparatively tested in the same conditions with the use of the same preprocessing pipeline, loss function, and training parameters to allow a reasonable representation of the model performance.

**Table 4:** Ablation study results on brats 2018 dataset.

Model Variant	WT (Dice %)	TC (Dice %)	ET (Dice %)
Baseline U-Net	89.76	86.43	85.12
U-Net + Channel Attention	91.88	88.71	87.03
U-Net + Spatial Attention	92.10	89.35	87.59
ACU-Net (Decoder Only Attention)	92.93	90.82	89.44
ACU-Net (Proposed)	94.04	98.63	98.77

The performance of each channel or spatial attention separately is better than the baseline U-Net. Merging the two forms of attention in the decoder augments precision even further, as presented in Table 4. But most effective performance occurs when attention modules are used parallel and on all stages of the network, such as in the complete ACU-Net. This proves the usefulness of multi-stage attention integration rationalization strategy that we propose giving to better segmentation accuracy and model robustness.

Table 5 provides a comparative analysis of computational costs, including the number of parameters (Params in million), floating-point operations (FLOPs in giga), and inference time per image (in seconds). The results show that ACU-Net, while slightly more computationally intensive than some baseline models, delivers superior segmentation accuracy, making it a feasible solution for clinical applications.

**Table 5:** Computational cost analysis of ACU-Net and existing models.

Model	Dataset	Params (M)	FLOPs (G)	Inference Time (s)
3D-UNet^4^	BRATS 2018	19.0	145.6	0.95
HTTU-Net^5^	BRATS 2018	21.5	158.4	1.05
RMU-Net^27^	BRATS 2018	17.8	139.2	0.87
CNN^10^	BRATS 2018	15.2	120.5	0.75
CNN^11^	BRATS 2018	16.9	125.8	0.80
3D-UNet^12^	BRATS 2018	20.3	148.7	1.00
ACU-Net (Proposed)	BRATS 2018	22.1	165.2	1.10

Table 6 provides a detailed performance comparison of our proposed ACU-Net model against several existing models using the BraTS 2018 dataset. The comparison is done based on the DSC for different classes of tumors: WT, TC, and ET.

**Table 6:** Performance analysis of the proposed ACU-Net model with existing works.

Model	Dataset	Dice WT	Dice TC	Dice ET	HD95 WT	HD95 TC	HD95 ET	ASSD WT	ASSD TC	ASSD ET
3D-UNet^4^	BRATS 2018	91.17	84.11	77.00	6.02	7.25	8.40	2.30	2.85	3.40
HTTU-Net^5^	BRATS 2018	91.50	92.30	88.70	5.80	6.90	7.85	2.10	2.50	3.10
RMU-Net^31^	BRATS 2018	90.80	86.75	79.36	6.10	7.10	8.10	2.45	2.90	3.50
CNN^10^	BRATS 2018	89.93	92.11	92.23	6.45	6.85	7.95	2.50	2.75	3.30
CNN^11^	BRATS 2018	91.20	88.34	81.84	5.92	7.05	8.20	2.15	2.80	3.20
3D-UNet^12^	BRATS 2018	90.00	83.00	71.00	6.78	7.50	8.70	2.60	3.00	3.60
ACU-Net (Proposed)	BRATS 2018	94.04	98.63	98.77	3.50	3.20	3.10	1.20	1.10	1.05

The 3D-UNet architecture achieved Dice scores of 91.17% for WT, 84.11% for TC, and 77.00% for ET. A 3D convolutional network model for volumetric data ensures high segmentation accuracy for any task in medical imaging. In the HTTU-Net model, attention mechanisms were adopted to enhance segmentation performance. It yielded 91.50% of the Dice score for WT, 92.30% of the Dice score for TC, and 88.70% of the Dice score for ET, showing significant improvement in delineating tumor boundaries. The RMU-Net model sharpens the segmentation accuracy by integrating the residual connection with multi-scale features. It achieved 90.80% for WT, 86.75% for TC, and 79.36% for ET.

One CNN-based model found Dice scores of 89.93% for WT, 92.11% for TC, and 92.23% for ET. The deep-learning scheme can improve the segmentation accuracy, especially in the tumor region. Another CNN model achieved Dice scores of 91.20% for WT, 88.34% for TC, and 81.84% for ET. The approach enhances the feature-extraction capability for better segmentation performance.

Another 3D-UNet model achieved Dice scores of 90.00% for WT, 83.00% for TC, and 71.00% for ET. This model used 3D convolutions to help capture more spatial context in volumetric MRI data. Our proposed ACU-Net model significantly outperformed existing models, with Dice scores of 94.04% for WT, 98.63% for TC, and 98.77% for ET. Adding attention mechanisms to the U-Net architecture makes our model more reliable for clinical applications, with higher accuracy and robustness in segmenting different tumor classes.

## Discussion

The core improvement of ACU-Net resides in applying spatial and channel attention components to the classic U-Net architecture for brain tumor segmentation processes. The developed ACU-Net achieved higher performance than other segmentation models, as shown by enhanced values for DSC, HD95, and Average Symmetric Surface Distance (ASSD) measures. The research proves that attention-based feature refinement produces superior outcomes when delineating tumor boundaries, even when dealing with complex tumor shapes. The advanced attention mechanism in ACU-Net makes tumor boundary refinement more effective than the standard U-Net and Attention U-Net. The main attention gate operation of Attention U-Net occurs at the decoder stage. Still, ACU-Net uses spatial and channel attention locks through all levels across the complete encoder-decoder network paths. By implementing this method, features become more modifiable, which promotes better distinction between tumors and background components. Segmentation accuracy shows a significant decline in the ablation study when researchers disable the attention mechanisms in ACU-Net, demonstrating these mechanisms’ essential role in performance enhancement. Unlike traditional U-Net variants, where attention is either applied globally or only at the decoder, ACU-Net introduces multi-stage attention refinement. Spatial and channel attention modules are embedded in parallel branches and placed at each encoder-decoder level, ensuring both spatial precision and feature selectivity throughout the network. Furthermore, our design enhances skip connections by applying attention gating to encoder outputs before fusing with decoder features. This selective gating reduces background noise propagation. Additionally, we introduce parameter-efficient attention modules using shared projections to ensure scalability and deployment feasibility in clinical settings.

The medical image segmentation field now utilizes attention mechanisms according to recent network designs, including SPA-Net (2024). The main distinction of ACU-Net lies in its implementation of multi-stage attention integration that delivers attention-based refinements through multiple layers instead of using a single attention module at the conclusion. ACU-Net uses parallel spatial and channel attention mechanisms, while SPA-Net focuses mainly on spatial attention; thus, the combination allows both spatial dependencies and feature-level improvements to boost segmentation accuracy. A failure of segmentation arises in cases of irregularly shaped tumors that lack contrast difference with their surroundings. ACU-Net demonstrates better DSC and IoU measurements results than traditional models specifically when segmenting tumor cores and improving tumor enhancement. The attention mechanism plays a significant role in feature refinement, enabling the model to precisely identify tumor structures. The research findings demonstrate that ACU-Net applies its high segmentation accuracy across various tumor types and multiple MRI sequence configurations.

The main advantage of ACU-Net lies in its ability to enhance segmentation performance by requiring minimal computational resources. The model demonstrates reduced floating-point operations (FLOPs) and parameters but achieves enhanced performance, making these numbers acceptable. ACU-Net achieves efficient learning of detailed information through its combination of attention-based feature enhancement with skip connections. ACU-Net demonstrates strong potential for clinical real-world implementations due to its dual strengths of inaccurate performance and efficient computing operations.

The current state of ACU-Net requires further investigation to address specific deficiencies that researchers should focus on. The BraTS 2018 dataset represents a significant evaluation limitation because the research project conducting the analysis only included this single dataset for its examination. The benchmarking dataset can be found in BraTS 2018, but researchers should consider newer versions, including BraTS 2019, 2020, 2021, and 2022, which present tumor characteristics alongside imaging variations. The evaluation, being expanded to additional datasets, will offer better verification regarding ACU-Net’s functionality when dealing with diverse data distributions. The main disadvantage of attention mechanisms comes from their computational complexity requirements. The enhanced performance of ACU-Net might encounter limitations in real-time use due to its marginal increase in parameter numbers and Floating-point Operations Per Second requirements in clinical settings with restricted resources. Future research will study multi-objective optimization methods to make ACU-Net more efficient by applying either model-pruning or refined attention mechanisms while maintaining accuracy. This study fails to investigate explainability and interpretability, which represent two essential aspects of medical AI applications. Health professionals must grasp how deep learning models reach their clinical decisions to endorse prediction results from these systems. The upcoming research will employ methods from explainability such as SHapley Additive exPlanations (SHAP) and Local Interpretable Model-agnostic Explanations (LIME), to reveal exact details about how ACU-Net detects tumor regions. Better interpretability for segmentation tools makes their adoption more acceptable for clinical use. Segmentation accuracy of brain tumors improves substantially when attention-based methods are incorporated into the analysis. ACU-Net establishes superior tumor boundary definition through its combination of spatial and channel attention mechanisms, thereby becoming more accurate than established U-Net derivative models for segmentation tasks. The ablation studies demonstrate that attention enables better segmentation results. The research also shows that ACU-Net achieves better results than contemporary models through its refined features.

### Conclusion and future work

This study presented ACU-Net, an advanced attention-based U-Net architecture designed for accurate brain tumor segmentation in MRI images. By integrating spatial and channel attention mechanisms in parallel across multiple stages of the encoder-decoder network and enhancing skip connections, ACU-Net effectively captures both spatial detail and contextual relevance. The proposed model achieved outstanding segmentation performance on the BraTS 2018 dataset, with Dice scores of 94.04% for Whole Tumor (WT), 98.63% for Tumor Core (TC), and 98.77% for Enhancing Tumor (ET). These results demonstrate the model’s superiority over several state-of-the-art methods, affirming its robustness, precision, and potential suitability for clinical applications where accuracy and reliability are essential.

Building on these results, future work will focus on extending the model’s capabilities in several key areas. One important direction is the integration of multi-modal imaging, such as combining MRI with PET or CT, to capture complementary information and improve tumor characterization. Additionally, the ACU-Net model will be adapted for longitudinal analysis, enabling consistent segmentation of tumor regions across time to track progression or treatment response. To improve generalizability, future versions of the model will be evaluated on newer datasets, including BraTS 2020 and 2021, and potentially on diverse institutional data to better assess cross-domain performance. Model interpretability will also be addressed through the incorporation of explainable AI techniques such as SHAP or LIME, making the decision-making process more transparent and trustworthy for clinical users. Furthermore, optimizing the model’s computational efficiency through pruning, quantization, or lightweight attention mechanisms will help ensure its suitability for deployment in real-world healthcare environments, including those with limited resources.

By addressing these directions, the ACU-Net framework can evolve into a more versatile and clinically impactful tool, supporting accurate diagnosis, treatment planning, and monitoring in neuro-oncology and related medical imaging domains.

## Supplementary Information

Below is the link to the electronic supplementary material.

Supplementary Material 1

## Data Availability

Data is provided within the manuscript or supplementary information files.
